# A Clinical Practice Framework for Ultrasound-Informed Multimodal Management of Post-Stroke Spasticity: A Proof-of-Concept Case

**DOI:** 10.3390/life16081271

**Published:** 2026-07-31

**Authors:** Emanuela Elena Mihai, Matei Teodorescu, Eugenio Moita Gonçalves, Rajiv Reebye, Mihai Berteanu

**Affiliations:** 1 Department of Physical and Rehabilitation Medicine, Carol Davila University of Medicine and Pharmacy Bucharest, 050474 Bucharest, Romania; 2 Department of Physical and Rehabilitation Medicine, Elias University Emergency Hospital, 011461 Bucharest, Romania; 3 Spectra LV Institute, Vancouver, BC V5Z 2G9, Canada; 4 Department of Physical Medicine and Rehabilitation, Hospital da Prelada, 4250-449 Porto, Portugal; 5 Division of Physical Medicine and Rehabilitation, Faculty of Medicine, University of British Columbia, Vancouver, BC V5Z 2G9, Canada

**Keywords:** neurological rehabilitation, stroke, spasticity, serial musculoskeletal ultrasound, multimodal approach, botulinum toxin type A (BoNT-A), physical modalities, neuromuscular electrical stimulation (NMES), extracorporeal shock wave therapy (ESWT)

## Abstract

Background: Stroke remains a leading cause of long-term disability worldwide. Post-stroke spasticity (PSS) is increasingly recognized as a complex complication resulting from the interaction between neural hyperexcitability and non-neural muscular adaptations, including fibrosis, altered muscle architecture, and increased stiffness. Consequently, effective management requires a comprehensive approach targeting both components. Musculoskeletal ultrasound (MSK US) has emerged as a practical tool for muscle characterization, treatment targeting, and longitudinal monitoring within neurorehabilitation programs. Aim: To propose a clinical practice framework integrating serial MSK US into multimodal PSS management and to illustrate its application through a proof-of-concept clinical case. Methods: The proposed framework combines standardized clinical assessment, serial MSK US evaluation, BoNT-A injections, physical modalities (neuromuscular electrical stimulation and extracorporeal shock wave therapy), and task-oriented rehabilitation. Ultrasound findings, including muscle echogenicity, architecture, and Modified Heckmatt Scale (MHS) characteristics, were integrated with clinical outcomes and rehabilitation goals to support individualized decision-making. Results: Serial MSK US provided complementary information regarding muscle quality and potential non-neural contributors to spasticity syndrome, supporting treatment planning and follow-up. Although only modest morphological changes were observed over time, the illustrative case showed favorable changes in clinical assessments including spasticity, motor performance, gait, and functional independence following the multimodal intervention. Conclusions: This proof-of-concept framework highlights the potential role of serial MSK US as a practical decision-support and monitoring tool within multimodal PSS management. Integrating clinical assessment with ultrasound-informed evaluation of both neural and non-neural components may facilitate more individualized rehabilitation strategies and optimize functional outcomes. Further studies are warranted to validate and standardize this approach.

## 1. Introduction

Stroke remains one of the leading causes of mortality and long-term disability worldwide [1,2,3]. Post-stroke spasticity (PSS) is a complex and dynamic syndrome that may substantially affect motor performance, activities of daily living, participation, and quality of life (QoL) [4]. Beyond velocity-dependent increases in muscle tone related to upper motor neuron lesions, PSS reflects the interaction between neural mechanisms, including reflex hyperexcitability, and non-neural mechanisms such as muscle shortening, fibrosis, altered viscoelastic properties, and connective tissue remodeling [5,6]. These structural adaptations may contribute to movement restriction, pain, and impaired function independently of neural overactivity and often become increasingly relevant in chronic stages after stroke [5,6].

The coexistence of neural and non-neural contributors makes spasticity syndrome management particularly challenging. Patients presenting with similar Modified Ashworth Scale scores may demonstrate markedly different muscle characteristics and treatment needs. Consequently, treatment failure or suboptimal response may not necessarily reflect inadequate pharmacological management but rather the presence of underlying muscle architectural changes that require complementary interventions. Recognition of these non-neural components has led to growing interest in assessment tools capable of characterizing muscle quality and supporting individualized treatment planning [5,6].

Musculoskeletal ultrasound (MSK US) has emerged as a practical, non-invasive bedside imaging modality capable of providing real-time information on muscle architecture, echogenicity, thickness, pennation patterns, and overall muscle quality [7,8]. Among the available ultrasound assessment methods, the Modified Heckmatt Scale (MHS) was introduced in 2020 as an adaptation of the original Heckmatt scale and provides a practical semi-quantitative evaluation of muscle echogenicity and structural alterations using conventional B-mode ultrasound [9,10]. The MHS has demonstrated good reliability and validity for assessing pathological muscle changes associated with spasticity and can be readily implemented in routine clinical practice without additional software or specialized equipment, making it particularly suitable for serial bedside assessment and longitudinal monitoring [9,10]. Unlike more advanced quantitative imaging techniques, the MHS can be readily implemented in routine clinical practice without additional software or specialized equipment, making it particularly suitable for serial bedside assessment.

Although ultrasound is widely used to improve the accuracy and safety of botulinum toxin type A (BoNT-A) injections, its role extends beyond procedural guidance. Serial musculoskeletal ultrasound may facilitate identification of chronic muscle changes, longitudinal monitoring of tissue adaptations, treatment targeting, and documentation of the non-neural component of post-stroke spasticity, thereby supporting individualized treatment planning [7,8,11,12,13,14,15]. Consequently, ultrasound has the potential to serve not only as an imaging modality but also as a practical clinical decision-support tool and a biomarker of muscle health and therapeutic response within multimodal neurorehabilitation programs [15,16,17,18,19].

Botulinum toxin type A (BoNT-A) remains the reference standard for focal spasticity management and represents a cornerstone of contemporary neurorehabilitation programs [15,16,17]. Nevertheless, optimization of treatment outcomes requires more than accurate muscle targeting. International recommendations increasingly emphasize goal-oriented and multimodal management strategies integrating BoNT-A with rehabilitation interventions tailored to the patient’s clinical presentation [15,16,17]. Adjunctive therapies such as neuromuscular electrical stimulation (NMES) and extracorporeal shock wave therapy (ESWT) have demonstrated potential benefits on muscle stiffness, pain, passive range of motion, and functional outcomes, particularly when incorporated into comprehensive rehabilitation programs [18,19,20,21,22,23,24,25,26].

The proposed framework is intended as an easily applicable approach for everyday clinical practice while also exploring the potential role of ultrasound as a monitoring and decision-support tool in future spasticity research. The aim of this article is to propose a practical ultrasound-informed clinical framework for post-stroke spasticity management integrating serial musculoskeletal ultrasound, standardized clinical assessment, botulinum toxin type A, and multimodal rehabilitation interventions. Using an illustrative clinical case, we present a proof-of-concept demonstrating how ultrasound-informed assessment may support treatment planning, longitudinal monitoring, and documentation of the non-neural component of post-stroke spasticity. The proposed framework is intended as a practical approach for routine clinical practice while providing a foundation for future validation studies.

## 2. Materials and Methods

### 2.1. Ethics Statement

According to institutional policies, ethical approval was not required for the description of a single anonymized clinical case to present a proof-of-concept. The patient provided written informed consent for participation in the rehabilitation program and for the use of anonymized clinical data and images for scientific publication, in accordance with the Declaration of Helsinki. No experimental interventions outside standard clinical practice were performed.

### 2.2. Clinical Assessment

Clinical assessment was performed using standardized and internationally recognized outcome measures commonly applied in post-stroke spasticity (PSS) and neurorehabilitation. Spasticity severity was assessed using the Modified Ashworth Scale (MAS). Functional mobility and gait performance were evaluated using the Timed Up and Go test (TUG) and Functional Ambulation Categories (FACs). Independence and participation were assessed using the Barthel Index, Activities of Daily Living (ADL), and Instrumental Activities of Daily Living (IADL) scales. Sensorimotor impairment was evaluated using the Fugl–Meyer Assessment for Upper and Lower Extremity (FMA-UE and FMA-LE). In addition to quantifying disability, these assessments were used to characterize the clinical impact of both neural and non-neural manifestations of spasticity and to guide individualized treatment goals. Assessments were performed at baseline and repeated longitudinally to support treatment planning and iterative clinical decision-making within the proposed framework. No statistical analyses were performed, as the assessments were used descriptively to illustrate framework implementation.

### 2.3. Musculoskeletal Ultrasound (MSK US) Assessment

Musculoskeletal ultrasound examinations were performed as part of routine clinical assessment to evaluate muscle morphology, echogenicity, and tissue characteristics in muscles affected by spasticity. Serial examinations were conducted using a GE Venue Go R3 ultrasound system (GE Healthcare) equipped with an L12 linear-array transducer (6–13 MHz). B-mode imaging was performed using standardized acquisition parameters. Patient positioning, probe placement, depth, focal zone placement, and minimal transducer pressure were standardized according to predefined anatomical landmarks to facilitate reproducibility across serial evaluations. Gain was generally maintained between 45% and 55%, and the focal zone was positioned at or slightly below the center of the target muscle to optimize visualization while maintaining comparable image quality across serial examinations.

Ultrasound assessment focused on the characterization of muscle architecture, echotexture, and structural changes associated with chronic post-stroke spasticity. Muscle echogenicity and structural alterations were evaluated semi-quantitatively using the Modified Heckmatt Scale (MHS). MHS findings were interpreted alongside standardized clinical assessment to support individualized treatment planning and longitudinal monitoring and were not considered standalone outcome measures.

### 2.4. Multimodal Rehabilitation Framework

The proposed framework integrates pharmacological treatment with physical modalities and comprehensive neurorehabilitation, guided by repeated clinical and ultrasound assessment.

Within the proposed proof-of-concept framework, musculoskeletal ultrasound fulfils four complementary roles: (1) identification and targeting of muscles selected for botulinum toxin injections, (2) semi-quantitative assessment of muscle structural changes using the Modified Heckmatt Scale, (3) longitudinal monitoring and documentation of the non-neural component of the post-stroke spasticity syndrome to support individualized treatment planning and adaptation over time, and (4) support for individualized clinical decision-making within a multimodal rehabilitation program.

Botulinum toxin type A (BoNT-A) represented the core pharmacological intervention for focal spasticity management. Injection planning was informed by clinical examination and musculoskeletal ultrasound findings to support targeted selection and individualized treatment strategies.

Physical modalities, including electrical stimulation (ES), namely neuromuscular electrical stimulation (NMES) and extracorporeal shock wave therapy (ESWT), were incorporated as complementary interventions based on clinical presentation and rehabilitation goals. These modalities were applied within routine clinical practice and integrated into a broader neurorehabilitation program, including task-oriented training and functional exercises. The framework emphasizes iterative reassessment, allowing treatment components to be adapted over time according to clinical evolution.

Complementary therapies were selected according to clinical findings and ultrasound-derived information regarding muscle quality and potential non-neural involvement. The framework therefore combines interventions targeting neural overactivity (e.g., BoNT-A) with interventions directed toward secondary muscular and connective tissue changes (e.g., NMES and ESWT), thereby addressing the multidimensional nature of post-stroke spasticity syndrome.

The overall workflow of the proposed ultrasound-informed multimodal clinical framework is summarized in Table 1. The framework integrates standardized clinical assessment, serial musculoskeletal ultrasound, individualized treatment planning, multimodal rehabilitation, and longitudinal monitoring to support clinical decision-making in post-stroke spasticity management.

**Figure 1:**
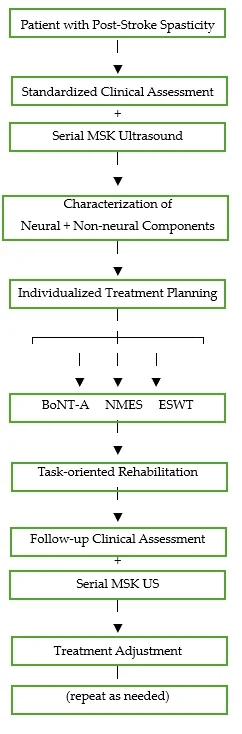
General neurorehabilitation flowchart. Abbreviations: BoNT-A: Botulinum toxin type A; ESWT: extracorporeal shock wave therapy; MSK US: musculoskeletal ultrasound; NMES: neuromuscular electrical stimulation.

The overall workflow of the proposed ultrasound-informed multimodal clinical framework is illustrated in Figure 1.

### 2.5. Conceptual Ultrasound-Informed Clinical Framework

**Figure 2:**
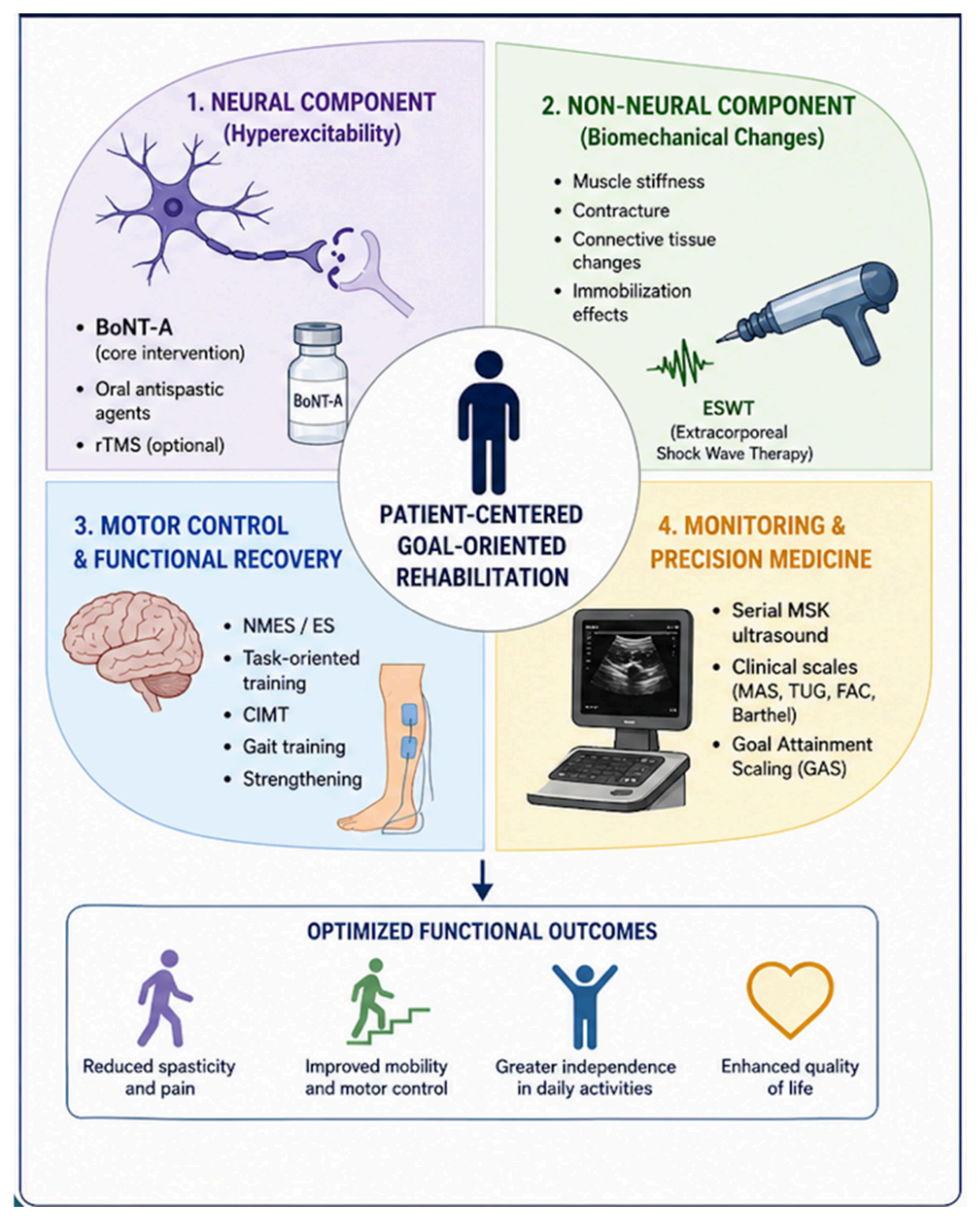
Conceptual ultrasound-informed clinical framework for multimodal management of post-stroke spasticity. The visual representation was initially generated using an AI-assisted tool and additionally manually refined and edited by the authors using standard software tools. The content is based on the core principles synthesized to highlight the proposed framework.

The conceptual foundations of the proposed ultrasound-informed multimodal clinical framework are illustrated in Figure 2. Whereas Figure 1 summarizes the sequential workflow of the framework, Figure 2 depicts the interactions between the neural and non-neural components of post-stroke spasticity, multimodal rehabilitation interventions, and longitudinal monitoring that underpin individualized clinical decision-making.

The framework is based on the concept that post-stroke spasticity represents a multidimensional syndrome resulting from the interaction between neural hyperexcitability and non-neural musculoskeletal adaptations, including changes in muscle architecture, stiffness, and tissue properties. Rather than representing a fixed treatment algorithm, the framework serves as a proof-of-concept model that integrates clinical assessment, serial musculoskeletal ultrasound, pharmacological interventions, physical agents, and functional rehabilitation to support personalized and adaptive management throughout the rehabilitation process.

## 3. Illustrative Clinical Case

To demonstrate the practical application of the proposed framework, a representative clinical case was selected from routine clinical practice. The case was chosen because it exemplified the coexistence of neural and non-neural manifestations of post-stroke spasticity and the need for individualized treatment decisions. Longitudinal clinical assessment and serial musculoskeletal ultrasound were used to guide and monitor a multimodal rehabilitation program incorporating ultrasound-guided BoNT-A injections, physical modalities (NMES and ESWT), and task-oriented neurorehabilitation. The case is presented as a proof-of-concept example illustrating how ultrasound-informed decision-making may be integrated into everyday clinical practice.

### 3.1. Patient Characteristics

A 52-year-old non-smoking male with cardiovascular risk factors, post-stroke spasticity, and right hemiparesis due to a hemorrhagic stroke was admitted to the Clinic of Neurological Rehabilitation, Elias University Emergency Hospital, Bucharest, Romania. Patient characteristics are summarized and presented in Table 2.

At admission, the patient presented right hemiparesis, facial hemihypoesthesia, central right facial hemiparesis, and spasticity grade three on the Modified Ashworth Scale (MAS) for the right upper and lower limbs. The patient also experienced moderate to high lower limb pain measured on the Visual Analogue Scale (VAS) of 7. In addition to clinically significant spasticity, the patient demonstrated important pain, reduced functional mobility, and evidence of chronic muscle alterations on musculoskeletal ultrasound, suggesting the coexistence of neural and non-neural contributors to movement impairment. Baseline musculoskeletal ultrasound demonstrated increased muscle echogenicity, heterogeneous echotexture, and partial loss of normal fascicular architecture, findings consistent with chronic post-stroke muscular adaptations. Semi-quantitative assessment using the Modified Heckmatt Scale corresponded predominantly to Grade 2 changes. Although severe structural alterations were not observed, the ultrasound findings, together with persistent pain, reduced tissue extensibility, and long-standing spasticity, suggested the presence of clinically relevant non-neural musculoskeletal components. These observations contributed to the decision to incorporate physical agent modalities, including ESWT and NMES, alongside BoNT-A and conventional neurorehabilitation.

The clinical examination was otherwise unremarkable. The patient’s past medical history was also significant for hypertension, dyslipidemia, and cardiac failure with left ventricular ejection fraction (LVEF) of 45%. His past surgical history was otherwise not significant.

The patient was chosen to illustrate the proposed framework through his medical history, hemiparesis, spasticity, functional mobility deficits, and recovery prognosis.

### 3.2. Baseline and Follow-Up Clinical and Msk Ultrasound Assessment

Clinical and musculoskeletal ultrasound (MSK US) assessments were performed at baseline (T0) and repeated at the 3-month follow-up (T1). Within the proposed framework, serial ultrasound findings were integrated with clinical examination to support treatment planning, monitor muscle characteristics over time, and guide individualized multimodal interventions. A summary of the integration of clinical and ultrasound findings within the decision-making process is presented in Table 3.

Clinical evaluation included spasticity severity assessed using the Modified Ashworth Scale (MAS), passive range of motion (PROM), pain intensity measured using the Visual Analogue Scale (VAS), sensorimotor impairment assessed with the Fugl–Meyer Assessment for Upper and Lower Extremity (FMA-UE and FMA-LE), functional ambulation evaluated using the Functional Ambulation Categories (FACs), and mobility assessed through the Timed Up and Go (TUG) test. Assessments were performed descriptively to illustrate framework implementation rather than treatment efficacy.

**Figure 3:**
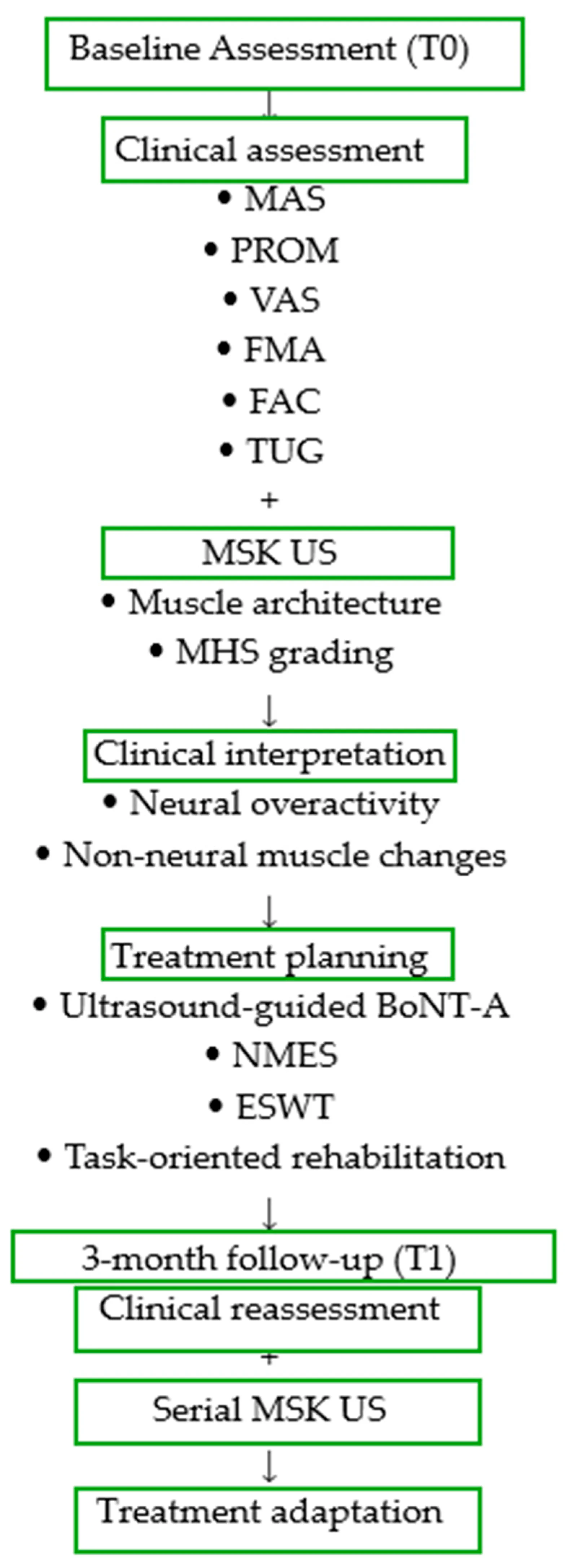
Application of the proposed ultrasound-informed multimodal clinical framework in the representative clinical case. Abbreviations: BoNT-A: Botulinum toxin type A; ESWT: extracorporeal shock wave therapy; FMA-LE: Fugl–Meyer Assessment for Lower Extremity; FMA-UE: Fugl–Meyer Assessment for Upper Extremity; MAS: Modified Ashworth Scale; MHS: Modified Heckmatt Scale; MSK US: musculoskeletal ultrasound; NMES: neuromuscular electrical stimulation; PROM: passive range of motion; TUG: Timed Up and Go Test; VAS: Visual Analogue Scale.

Following baseline clinical and musculoskeletal ultrasound assessment, the patient underwent an individualized multimodal rehabilitation program according to the proposed framework. The sequential rehabilitation strategy implemented in this illustrative case is summarized in Figure 3.

**Figure 4:**
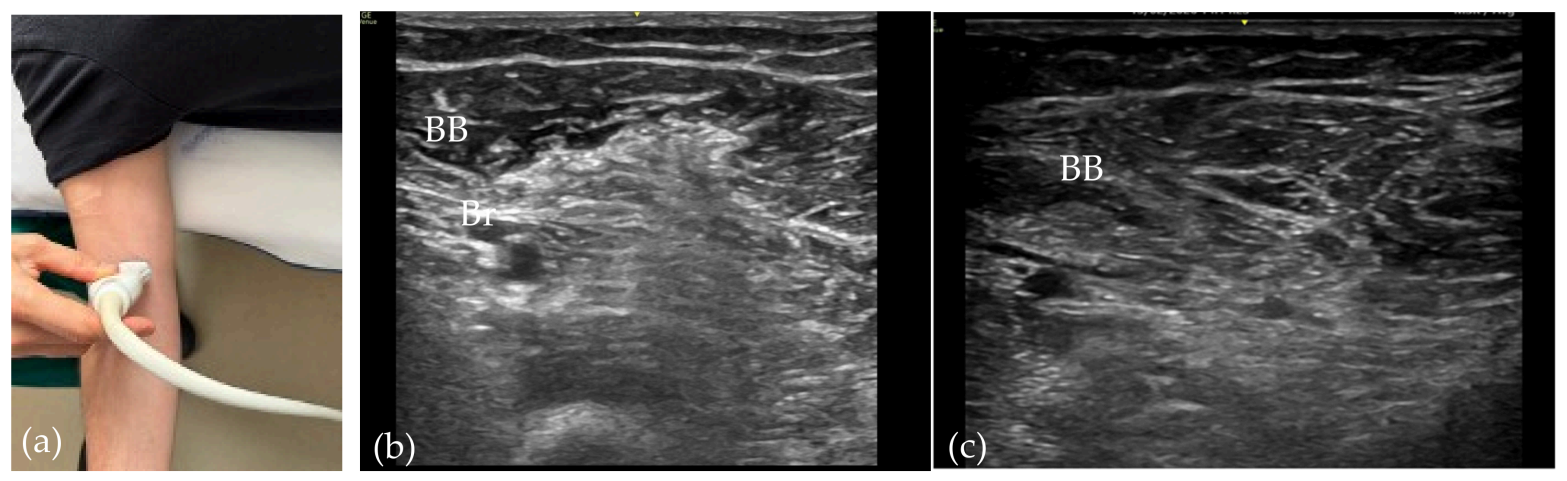
MSK US probe placement. BB, brachialis muscle and brachial artery assessed at T0 and T1. Abbreviations: BB: biceps brachii; MSK US: musculoskeletal ultrasound; T0: baseline assessment; T1:follow-up at 3 months; (**a**) Patient positioning and probe placement at the mid-arm level. (**b**) MSK US image of BB, brachialis muscle, and brachial artery at T0. Baseline ultrasound image demonstrating the superficial biceps brachii muscle and the deeper brachialis muscle adjacent to the brachial artery. Increased echogenicity and altered muscle architecture can be observed. (**c**) MSK US image of BB, brachialis muscle, and brachial artery at T1. Follow-up ultrasound image obtained at 3 months using the same acquisition protocol. Muscle architecture remained largely preserved, with only subtle changes in echotexture despite clinically meaningful functional improvement.

**Figure 5:**
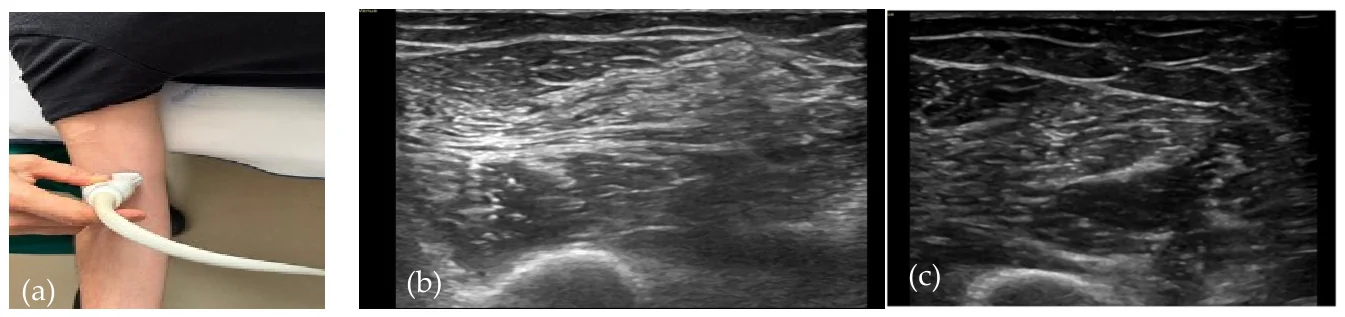
MSK US probe placement and brachialis muscle. Abbreviations: MSK US: Musculoskeletal ultrasound; T0: baseline assessment; T1: follow-up at 3 months; (**a**) Patient positioning and probe placement at the distal third of the arm in transverse orientation. (**b**) MSK US image of brachialis muscle at T0. Baseline ultrasound image demonstrating the brachialis muscle located between the humeral cortex and the overlying biceps brachii muscle. (**c**) MSK US of brachialis muscle at T1. Follow-up ultrasound image obtained at 3 months using the same acquisition protocol. Muscle architecture remained largely unchanged, with persistent heterogeneous echotexture and only subtle differences compared with baseline.

**Figure 6:**
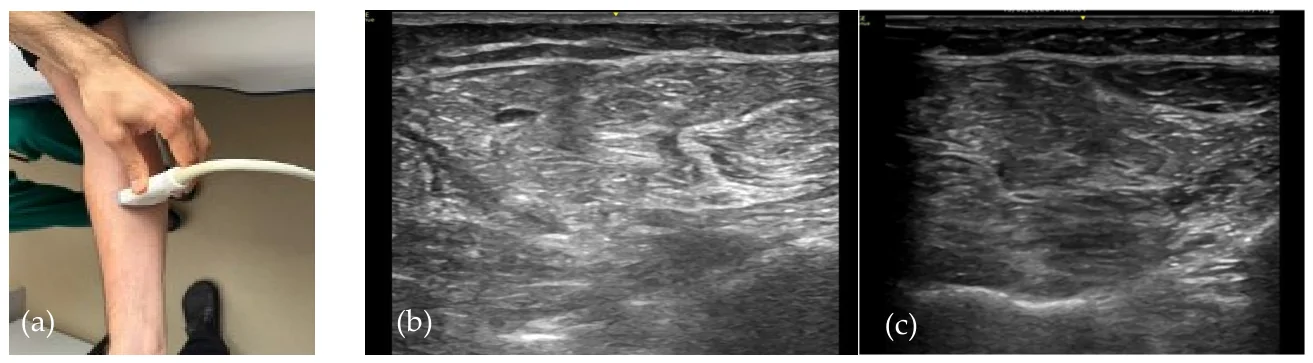
MSK US probe placement. PL, FCR, PT muscles. (**a**) Patient positioning and probe placement at the proximal third of the forearm in transverse orientation. (**b**) MSK US image of PL, FCR, PT muscles at T0. Baseline ultrasound image demonstrating the palmaris longus, flexor carpi radialis, and pronator teres muscles. Increased echogenicity and reduced differentiation between muscle boundaries can be observed, consistent with chronic muscular adaptations associated with post-stroke spasticity. The flexor carpi radialis was selected as a target muscle for ultrasound-guided BoNT-A injection. (**c**) MSK US image of PL, FCR, PT muscles at T1. Follow-up ultrasound image obtained at 3 months using the same acquisition protocol. Persistent architectural alterations remained visible, although a slight reduction in tissue heterogeneity was observed. Overall muscle morphology remained largely unchanged compared with baseline. Abbreviations: FCR: flexor carpi radialis; MSK US: Musculoskeletal ultrasound; PL: Palmaris longus; PT: pronator teres; T0: baseline assessment; T1: follow-up at 3 months.

**Figure 7:**
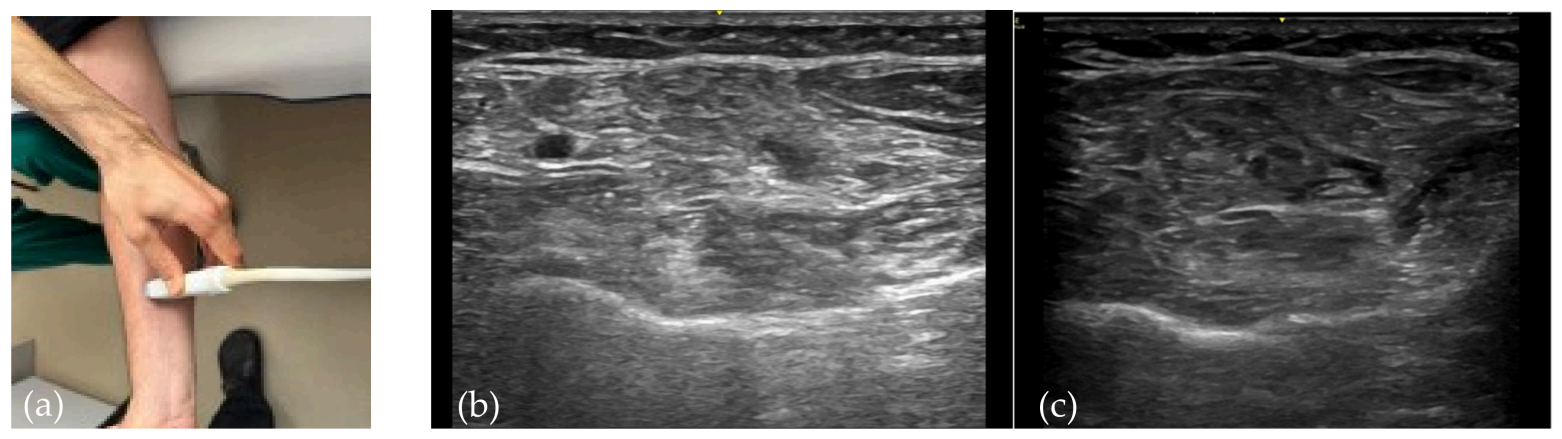
MSK US probe placement. Flexor digitorum superficialis (FDS) and flexor digitorum profundus (FDP). Abbreviations: FDP: Flexor digitorum profundus; FDS: Flexor digitorum superficialis; MSK US: Musculoskeletal ultrasound; T0: baseline assessment; T1: follow-up at 3 months. (**a**) Patient positioning and probe placement at the mid-forearm level in transverse orientation. (**b**) MSK US image of FDS and FDP muscles at T0. Baseline ultrasound image demonstrating the flexor digitorum superficialis (FDS) and flexor digitorum profundus (FDP) muscles separated by the intermuscular fascia. The median nerve is visible between the two muscle groups and serves as an important anatomical landmark during ultrasound-guided injection procedures. (**c**) MSK US image of FDS and FDP muscles at T1. Follow-up ultrasound image obtained at 3 months using the same acquisition protocol. Muscle architecture remained generally stable, with persistent echogenic changes and only subtle differences compared with baseline assessment.

**Figure 8:**
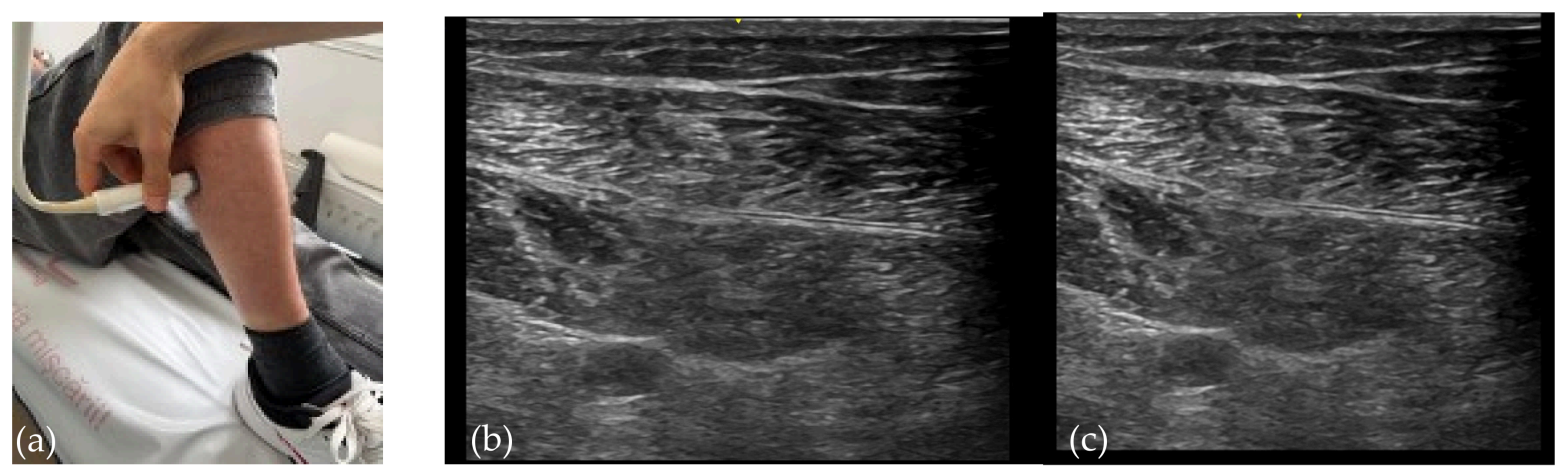
MSK US probe placement. MG, LG, and soleus muscle. (**a**) Follow-up ultrasound image obtained at 3 months using the same acquisition protocol. Persistent chronic muscle changes remained evident, with only minor differences in echotexture and overall muscle appearance. These muscles were selected for multimodal intervention based on the combined interpretation of clinical findings, pain presentation, functional limitations, and ultrasound characteristics. Abbreviations: LG: Lateral head of gastrocnemius; MG: Medial head of gastrocnemius; MSK US: Musculoskeletal ultrasound; T0: baseline assessment; T1: follow-up at 3 months.

**Figure 9:**
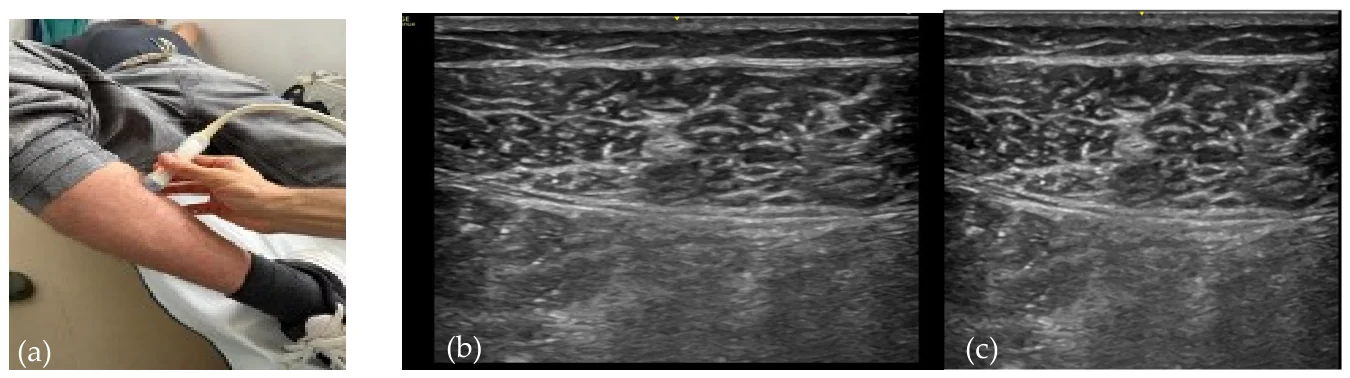
MSK US probe placement. MG, LG, and soleus muscle. (**a**) Patient positioning and probe placement for visualization of the triceps surae complex. (**b**) MSK US image of MG, LG, and soleus muscle at T0. Baseline ultrasound image demonstrating the soleus muscle located beneath the medial and lateral heads of the gastrocnemius muscle. Increased echogenicity and heterogeneous echotexture are visible, suggesting chronic structural adaptations associated with post-stroke spasticity. This view allows reliable identification of the triceps surae complex while minimizing the risk of inadvertent injection of adjacent structures. (**c**) MSK US image of MG, LG, and soleus muscle at T1. Follow-up ultrasound image obtained at 3 months using the same acquisition protocol. Muscle architecture remained largely preserved, with subtle improvements in echotexture but no major structural changes. These muscles were selected for multimodal intervention based on the combined interpretation of clinical findings, pain presentation, functional limitations, and ultrasound characteristics. Abbreviations: LG: Lateral head of gastrocnemius; MG: Medial head of gastrocnemius; MSK US: Musculoskeletal ultrasound; T0: baseline assessment; T1: follow-up at 3 months.

**Figure 10:**
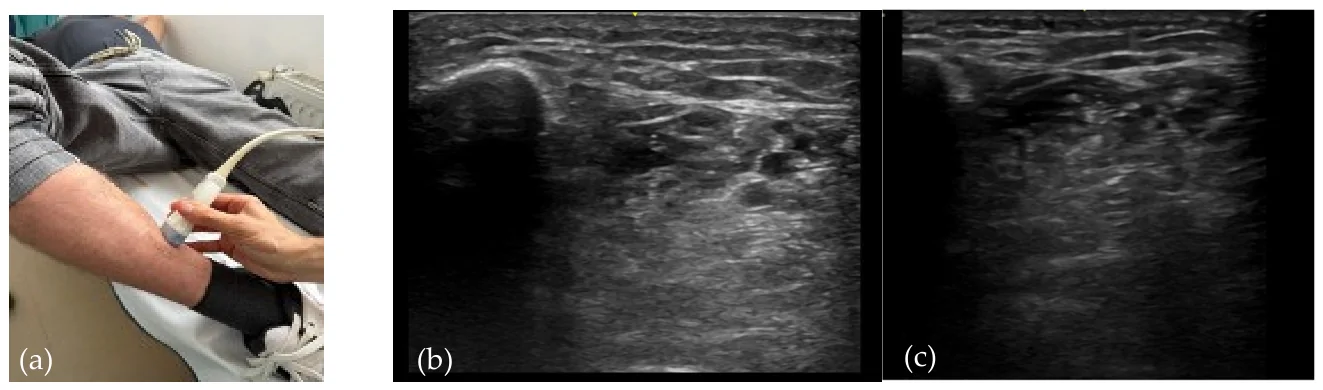
MSK US probe placement. Tibialis posterior muscle. (**a**) Patient positioning and probe placement at the distal third of the calf in cross-sectional plan. (**b**) MSK US image of tibialis posterior muscle at T0. Baseline ultrasound image demonstrating the tibialis posterior (TP), flexor hallucis longus (FHL), and flexor digitorum longus (FDL) muscles. Due to chronic post-stroke muscular changes, differentiation of individual muscle boundaries may be challenging, highlighting the importance of dynamic and ultrasound-guided assessment. (**c**) MSK US image of tibialis posterior muscle at T1. Follow-up ultrasound image obtained at 3 months using the same acquisition protocol. Persistent increases in echogenicity and architectural alterations remained visible, although slight improvement in muscle echotexture was observed. Overall structural changes remained relatively stable compared with baseline. These muscles were selected for multimodal intervention based on the combined interpretation of clinical findings, pain presentation, functional limitations, and ultrasound characteristics. Abbreviations: MSK US: Musculoskeletal ultrasound; T0: baseline assessment; T1: follow-up at 3 months.

Serial musculoskeletal ultrasound examinations were performed according to the standardized acquisition protocol described in the Section 2. Representative baseline and follow-up images are presented in Figure 4, Figure 5, Figure 6, Figure 7, Figure 8, Figure 9 and Figure 10.

At the 3-month follow-up, ultrasound demonstrated only modest changes in muscle morphology compared with baseline. Persistent increases in echogenicity and architectural alterations remained evident in several muscles, although subtle improvements in muscle texture and fascicular definition were observed in selected regions. These imaging findings contrasted with the more pronounced improvements observed in spasticity, pain, mobility, sensorimotor performance, and functional independence. Consequently, ultrasound findings were interpreted in conjunction with clinical outcomes rather than as isolated indicators of treatment response.

Serial musculoskeletal ultrasound complemented clinical assessment by supporting treatment planning, longitudinal monitoring, and individualized adaptation of the rehabilitation program.

### 3.3. Multimodal Neurological Rehabilitation Program

Within the proposed framework, post-stroke spasticity was approached as a multidimensional condition resulting from the interaction between neural hyperexcitability and secondary musculoskeletal adaptations. Consequently, management combined pharmacological, physical, and rehabilitation interventions targeting both components.

The conventional neurological rehabilitation program consisted of individualized task-oriented therapy, active and passive range-of-motion exercises, stretching, balance and gait training, functional training, and the use of assistive devices and orthoses when required. Rehabilitation goals were established according to the patient’s functional limitations and periodically reassessed throughout the intervention period.

Physical modalities were incorporated to complement BoNT-A treatment and address pain, muscle stiffness, and potential non-neural contributors to spasticity. Radial extracorporeal shock wave therapy (rESWT; Endopuls 811, Enraf Nonius B.V., Rotterdam, The Netherlands) was applied at the gastrocnemius–soleus myotendinous junction using 2000 pulses, a frequency of 10 Hz, and an energy density of 1 bar. Two treatment sessions were performed over a two-week period. The intervention was well tolerated, with no adverse events reported.

Neuromuscular electrical stimulation (NMES) was applied to selected antagonist muscle groups to facilitate motor recruitment, support voluntary activation, and complement the pharmacological effects of BoNT-A. Stimulation parameters ranged between 25 and 50 Hz and 200–400 μs pulse duration, with intensity adjusted to achieve visible muscle contraction while maintaining patient comfort. Sessions were performed daily during hospitalization for a total of 10 treatment sessions. No adverse effects were reported.

The selection of physical agent modalities was based on the combined interpretation of clinical findings, rehabilitation goals, pain presentation, and musculoskeletal ultrasound characteristics, thereby supporting an individualized multimodal treatment strategy.

### 3.4. BoNT-A Injection Strategy

BoNT-A represented the core pharmacological intervention within the proposed multimodal framework. Muscle selection and dose allocation were based on a combination of clinical examination, functional goals, and musculoskeletal ultrasound findings, with the objective of addressing the neural component of post-stroke spasticity while facilitating subsequent rehabilitation interventions.

Injections were performed under sterile conditions using ultrasound guidance throughout the procedure. A 25–27-gauge needle was used, and the toxin was reconstituted with preservative-free 0.9% saline to achieve an appropriate dilution for individualized dose distribution. Real-time ultrasound imaging was used to identify target muscles, guide needle placement, confirm intramuscular localization, and avoid adjacent neurovascular structures.

The selected upper-limb muscles included the pectoralis major, biceps brachii, brachialis, pronator teres, flexor carpi radialis, and flexor digitorum superficialis. Lower-limb targets included the gastrocnemius, soleus, and tibialis posterior muscles. Ultrasound assessment supported accurate identification of both superficial and deep muscle structures and contributed to individualized treatment planning, particularly in muscles demonstrating chronic architectural alterations associated with spasticity.

The complete injection strategy, including muscle selection and dose distribution, is presented in Table 4. Within the proposed framework, ultrasound-guided BoNT-A injections were integrated with physical agent modalities and task-oriented rehabilitation to address both neural and non-neural contributors to movement impairment and functional limitation.

The rationale for combining BoNT-A, NMES, and ESWT was based on the hypothesis that simultaneously addressing neural overactivity and secondary musculoskeletal adaptations may optimize functional outcomes beyond pharmacological treatment alone for a patient who was suffering from continuous pain.

### 3.5. Integration of Clinical and Ultrasound Findings in Longitudinal Management

Longitudinal assessment was performed to monitor clinical evolution and support iterative decision-making within the proposed framework. Changes in spasticity, pain, sensorimotor performance, mobility, ambulation, and functional independence were evaluated using standardized outcome measures, including the Modified Ashworth Scale (MAS), Visual Analogue Scale (VAS), Fugl–Meyer Assessment (FMA-UE and FMA-LE), Functional Ambulation Categories (FACs), Timed Up and Go (TUG), Activities of Daily Living (ADL), and Instrumental Activities of Daily Living (IADL).

At the 3-month follow-up, improvements were observed across several clinical domains, including spasticity severity, mobility, ambulation, motor performance, pain, and functional independence. Although serial musculoskeletal ultrasound demonstrated only modest changes in muscle architecture and echotexture, these findings were interpreted alongside the more pronounced clinical and functional improvements. This observation supports the concept that post-stroke spasticity reflects the interaction of neural and non-neural mechanisms, which may evolve differently over time.

Within the proposed framework, serial musculoskeletal ultrasound was not used as a standalone outcome measure but rather as a complementary tool to support muscle targeting, monitor muscle quality, and guide individualized treatment adaptation. Clinical outcomes are summarized in Table 3 previously presented.

**Figure A1:**
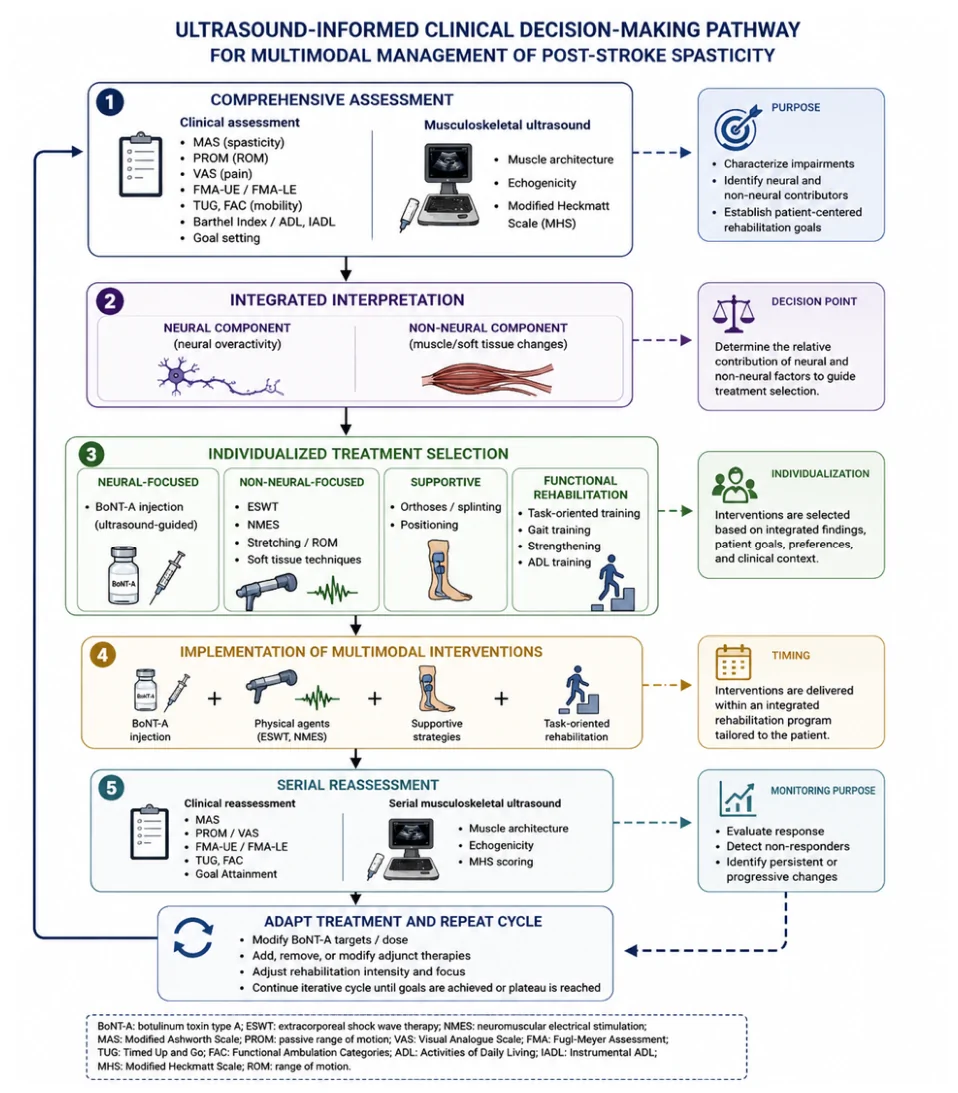
**Ultrasound-informed clinical decision-making algorithm for multimodal management of post-stroke spasticity.** The algorithm integrates standardized clinical assessment and serial musculoskeletal ultrasound to support individualized treatment planning, implementation of multimodal interventions, longitudinal monitoring, and adaptive treatment modification according to the relative contribution of neural and non-neural components of spasticity. The visual representation was initially generated using an AI-assisted tool and additionally manually refined and edited by the authors using standard software tools. The content is based on the core principles synthesized to highlight the proposed framework and guide evidence-based decisions for optimal results.

To further illustrate the practical application of the proposed framework, Figure A1 (Appendix A) presents an ultrasound-informed clinical decision-making algorithm for multimodal management of post-stroke spasticity. Building upon the conceptual framework presented in Figure 1, the algorithm demonstrates how standardized clinical assessment and serial musculoskeletal ultrasound can be integrated to support individualized treatment planning, implementation of multimodal interventions, longitudinal monitoring, and adaptive treatment modification. By emphasizing the complementary evaluation of neural and non-neural contributors to spasticity, the algorithm provides a practical model for implementing the proposed framework in routine neurorehabilitation practice.

## 4. Discussion

### 4.1. Principal Findings

The present report should be interpreted as a proof-of-concept illustrating the feasibility of an ultrasound-informed clinical practice framework rather than as evidence of treatment efficacy. The objective was not to demonstrate the effectiveness of a specific intervention, but to show how serial musculoskeletal ultrasound, interpreted alongside standardized clinical assessment, may support treatment planning, longitudinal monitoring, and documentation of the non-neural component of post-stroke spasticity within routine rehabilitation practice. While PSS reflects the interaction of neural and non-neural mechanisms, the principal focus of the proposed framework is the systematic assessment and longitudinal monitoring of the non-neural musculoskeletal component using serial ultrasound, interpreted in conjunction with standardized clinical assessment. Objective neurophysiological techniques may further characterize neural mechanisms and represent an important direction for future validation studies but were beyond the scope of this proof-of-concept, which was intentionally designed as a pragmatic framework suitable for routine clinical practice. PSS is increasingly recognized as a condition evolving over time through the interaction of altered neural control and secondary musculoskeletal adaptations [4]. Beyond velocity-dependent hypertonia, changes in muscle architecture, connective tissue, and mechanical properties may substantially influence clinical presentation and response to treatment [15]. These non-neural components are particularly relevant in the subacute and chronic phases after stroke and may limit functional recovery if not adequately addressed [4,15].

PSS represents a common complication after stroke and can contribute significantly to disability and secondary complications if not recognized and managed appropriately. Early identification and targeted treatment are therefore essential to prevent contractures, abnormal movement patterns, and functional decline. Botulinum toxin type A, combined with rehabilitation interventions, plays an important role in the management of post-stroke spastic movement disorders within a comprehensive neurorehabilitation strategy [16]. Recognizing spasticity as a dynamic, multidimensional condition supports the need for repeated assessment and individualized treatment strategies [4,15].

BoNT-A is widely recognized as the standard of care for the treatment of focal spasticity following stroke. By reducing muscle overactivity, BoNT-A represents a key pharmacological intervention within multidisciplinary neurorehabilitation programs [17]. However, clinical outcomes following BoNT-A treatment are influenced by multiple factors, including muscle selection, dosing, timing, and integration with rehabilitation therapies [8]. Evidence highlighted the clinical benefits of botulinum toxin injections guided by imaging techniques. For example, Çelebi et al. [27] demonstrated that ultrasound-guided BoNT-A injections significantly reduced spasticity and pain while improving upper limb motor function in patients with post-stroke spasticity, supporting the role of imaging in optimizing injection accuracy and functional outcomes. The present framework emphasizes BoNT-A as a central intervention whose clinical impact may be optimized when embedded within a structured, assessment-driven, multimodal rehabilitation strategy. Other studies have explored different guidance techniques for BoNT-A injections. Hauret et al. [28] compared ultrasound-guided and electrical-stimulation-guided injections for triceps surae spasticity and found no significant differences in clinical outcomes, although ultrasound guidance allowed faster procedures. These findings suggest that multiple targeting techniques may be effective, while ultrasound provides additional advantages in visualization and procedural efficiency [28].

Musculoskeletal ultrasound is increasingly recognized not only as a guidance technique for botulinum toxin injections but also as a potential clinical biomarker of muscle health [7]. In post-stroke spasticity, ultrasound can provide complementary information regarding muscle architecture, echogenicity, fascicular organization, and tissue quality, thereby offering insights into the non-neural component of the spasticity syndrome [7]. Within the proposed framework, serial MSK US served as both a targeting and monitoring tool, supporting individualized treatment planning and longitudinal reassessment. Although standardized ultrasound-based decision-making algorithms remain under development, the integration of imaging findings with clinical assessment may facilitate more precise and adaptive management strategies in routine neurorehabilitation practice.

The illustrative case provided a practical example of how serial ultrasound findings can be integrated into clinical decision-making. Baseline imaging demonstrated persistent increases in echogenicity, heterogeneous muscle texture, and architectural alterations consistent with chronic post-stroke muscular adaptations. Follow-up ultrasound demonstrated only modest changes despite improvements in pain, spasticity, mobility, and functional performance. This apparent discrepancy reinforces an important concept underlying the proposed framework: clinical recovery and structural muscle remodeling do not necessarily occur in parallel. Considering the pathophysiology of non-neural components, there are changes at the extracellular matrix level and hyaluronan accumulation. The non-neural component is driven primarily by changes in the muscle extracellular matrix (ECM) [29]. Hyaluronan (HA), a glycosaminoglycan, accumulates in spastic muscles and undergoes biophysical alterations that increase ECM viscosity, resulting in increased passive mechanical resistance. Elevated hyaluronan disrupts the viscoelastic and lubricating properties of muscle tissue, increasing friction and impairing fiber gliding during movement. Additionally, over time, hyaluronan aggregation may induce fibroblast-to-myofibroblast transformation, leading to irreversible muscle fibrosis and joint contractures. Subclinical contractures that contribute to passive stiffness. Nonetheless, even though structural changes on MSK US were not markedly pronounced, the patient demonstrated clear clinical and functional improvement during the rehabilitation program. These findings highlight the complementary role of ultrasound within the proposed framework, supporting muscle identification and treatment planning while clinical and functional outcomes remain central to evaluating therapeutic response. While these findings are encouraging, they should not be interpreted as evidence of efficacy because they derive from a single patient and primarily serve to demonstrate the application of the proposed framework.

Complementary physical modalities such as neuromuscular electrical stimulation (NMES) and extracorporeal shock wave therapy (ESWT) have been increasingly explored for their potential effects on muscle tone modulation, tissue properties, and functional outcomes in post-stroke spasticity [19,20,21,22]. Physical modalities have also been investigated as complementary strategies to enhance the effects of botulinum toxin. Lee and Yang reported that the addition of extracorporeal shock wave therapy following BoNT-A injections improved spasticity outcomes in patients with post-stroke upper limb spasticity, supporting the concept of multimodal management strategies [18]. Evidence from systematic reviews also supports the effectiveness of both botulinum toxin injections and shock wave therapy in reducing spasticity. A network meta-analysis including more than 1900 patients demonstrated significant improvements in spasticity scores following both interventions, highlighting the potential complementary role of pharmacological and physical modalities in spasticity management [23].

Within the proposed framework, these physical modalities are positioned as complementary interventions rather than alternatives to BoNT-A. Their integration is guided by clinical presentation, functional goals, and ultrasound findings, with the aim of addressing secondary musculoskeletal changes and facilitating task-oriented rehabilitation. This multimodal perspective supports a comprehensive approach to spasticity management that extends beyond pharmacological treatment alone.

### 4.2. Practical Implications of the Proposed Framework

The practical value of the proposed framework lies in its feasibility and adaptability to routine neurorehabilitation practice. Rather than introducing a novel intervention, the framework integrates established assessment tools and therapeutic modalities into a structured, goal-oriented pathway that can support individualized clinical decision-making. Serial clinical assessment, musculoskeletal ultrasound, ultrasound-guided BoNT-A injections, adjunctive therapies, and task-oriented rehabilitation are already available in many rehabilitation settings. Consequently, the framework provides clinicians with a pragmatic model for systematically addressing both neural and non-neural contributors to post-stroke spasticity syndrome while facilitating longitudinal treatment optimization.

The proposed framework has several potential clinical implications. First, it encourages a shift from single-intervention strategies toward integrated, reassessment-driven care pathways. Second, it highlights the potential role of musculoskeletal ultrasound as a tool for refining BoNT-A treatment and guiding adjunctive therapies. Future research should focus on validating standardized ultrasound protocols, exploring optimal sequencing of multimodal interventions, and assessing the impact of this framework in larger patient cohorts. Such efforts may further advance evidence-based, personalized approaches to spasticity management.

From a clinical perspective, the proposed framework may also facilitate quality improvement within specialized spasticity services. Standardized acquisition of serial musculoskeletal ultrasound images, interpreted alongside clinical and functional outcome measures, provides a structured method for documenting muscle health, monitoring treatment response, and adapting individualized rehabilitation strategies over time. Such an approach may improve consistency of care and support benchmarking of multidisciplinary spasticity management programs. Nevertheless, musculoskeletal ultrasound should not be viewed solely as a guidance tool for injections, but as a practical semi-quantitative clinical decision-support tool for targeting, monitoring, and documenting the non-neural component of the post-stroke spasticity syndrome within a multimodal rehabilitation framework.

### 4.3. Clinical Implications-Why Improvements Occur Despite Minimal Sonographic Changes

Our findings regarding pain reduction and functional improvements occurred despite non-crucial sonographic changes and align with current evidence:

• Botulinum toxin primarily targets neural mechanisms by blocking acetylcholine release at the neuromuscular junction, reducing reflex-mediated spasticity. However, it does not directly address the non-neural components of muscle stiffness and fibrosis [5].
• ESWT may modulate both components through multiple mechanisms: modulating muscle rheology and viscoelastic properties; potentially affecting neurotransmission at the neuromuscular junction; improving tissue extensibility and reducing passive stiffness; combined therapy (BoNT-A + ESWT) shows superior and more prolonged spasticity reduction compared to BoNT-A alone, with improvements lasting up to 25–26 weeks [19,20,21,23,25].
• NMES effects on non-neural components: Neuromuscular electrical stimulation can improve range of motion and reduce spasticity through both neural modulation and mechanical effects on muscle tissue [26,30].
• Pain and function improvements may precede structural changes: clinical improvements in pain, range of motion, and function can occur through reduced reflex-mediated muscle overactivity; improved muscle fiber gliding despite persistent ECM changes; enhanced proprioception and motor control; reduced inflammation and nociceptive input.

Beyond monitoring treatment response, musculoskeletal ultrasound may assist in identifying muscles that could benefit from adjunctive interventions targeting the non-neural component of spasticity [8]. Increased echogenicity, altered fascicular organization, and evidence of chronic muscle remodeling may suggest the presence of secondary biomechanical changes that are less likely to respond to pharmacological treatment alone. In such situations, adjunctive interventions including ESWT, NMES, stretching, and task-oriented rehabilitation may be considered as part of a multimodal management strategy [15]. Although this concept requires prospective validation, it represents one of the key hypotheses underpinning the proposed framework.

While the proposed framework acknowledges that post-stroke spasticity results from the interaction of neural and non-neural mechanisms, its principal focus is the practical assessment and longitudinal monitoring of the non-neural musculoskeletal component using serial musculoskeletal ultrasound interpreted alongside standardized clinical assessment. Objective neurophysiological techniques, including surface electromyography and somatosensory evoked potentials, may provide complementary information regarding the neural component of spasticity and have shown promise for quantifying neural dysfunction and monitoring neurophysiological changes following stroke [31,32]. However, these techniques were beyond the scope of the present proof-of-concept, which was intentionally designed as a pragmatic clinical framework that can be readily implemented using assessment methods commonly available in routine rehabilitation practice. Future prospective studies integrating objective neurophysiological assessments with serial musculoskeletal ultrasound may provide a more comprehensive characterization of the neural and non-neural components of post-stroke spasticity and further validate the proposed framework.

An important concept highlighted by the proposed framework is the distinction between adjunctive and multimodal therapy in post-stroke spasticity management. Although these terms are often used interchangeably, they represent different therapeutic strategies [15]. Adjunctive therapy refers to the addition of a secondary intervention specifically intended to enhance the effect of a primary treatment within a defined time window, such as the application of neuromuscular electrical stimulation, casting, or extracorporeal shock wave therapy following BoNT-A injections. In contrast, multimodal therapy represents a broader patient-centered rehabilitation strategy that combines pharmacological and non-pharmacological interventions in a coordinated and individualized manner to address the diverse neural and non-neural contributors to spasticity. Within this model, adjunctive therapies may serve as components of a multimodal rehabilitation program, supporting the effects of a specific intervention while contributing to broader functional goals [15]. Understanding this distinction is important for treatment planning, as successful spasticity management often requires both optimization of individual interventions and integration of complementary therapies within a comprehensive rehabilitation pathway [15,17].

An additional consideration is the timing of adjunctive interventions relative to BoNT-A administration. Current evidence suggests that adjunctive therapies intended to enhance the effects of BoNT-A, such as NMES and ESWT, should ideally be initiated shortly after injection, typically within the first few days and up to approximately two weeks following treatment [15,33,34]. Nevertheless, published recommendations and clinical practice guidelines acknowledge variability in treatment scheduling and implementation across rehabilitation settings [35]. Within the proposed framework, adjunctive therapies are not viewed as isolated interventions but rather as integrated components of a broader multimodal rehabilitation strategy. Accordingly, NMES and ESWT were incorporated following BoNT-A administration to address both neural and non-neural contributors to post-stroke spasticity, while simultaneously supporting functional recovery through task-oriented rehabilitation. This integrated approach reflects the concept that adjunctive therapies may optimize the effect of a specific intervention, whereas multimodal rehabilitation aims to address the complex and evolving nature of the spasticity syndrome as a whole.

A central concept of the proposed framework is the use of musculoskeletal ultrasound not only for treatment targeting but also for longitudinal monitoring of the non-neural component of the post-stroke spasticity syndrome. Serial imaging allows documentation of muscle architecture, echogenicity, and structural adaptations over time, providing complementary information that may not be captured by clinical scales alone. Although substantial architectural remodeling was not observed during the relatively short follow-up period in the present case, ultrasound findings contributed to treatment planning, supported the selection of interventions, and facilitated documentation of muscle status throughout rehabilitation. In this context, semi-quantitative tools such as the Modified Heckmatt Scale may represent practical methods for integrating ultrasound findings into routine clinical decision-making.

This framework also reflects the growing recognition that successful spasticity management extends beyond the reduction in muscle tone alone. Clinical decision-making should consider the complex interaction between neural hyperexcitability, secondary musculoskeletal changes, motor impairment, activity limitations, and patient-centered goals. By integrating serial clinical assessment and musculoskeletal ultrasound with BoNT-A, adjunctive physical modalities, and task-oriented rehabilitation within a unified pathway, the proposed model encourages a more comprehensive and individualized approach to post-stroke spasticity management. In this context, musculoskeletal ultrasound serves not only as a guidance technique for injections but also as a complementary tool for muscle characterization, treatment planning, and longitudinal monitoring. Such an approach may facilitate the translation of current evidence into daily practice while providing a foundation for future studies investigating imaging-guided and multimodal treatment strategies. An additional aspect illustrated by the proposed framework is the potential role of semi-quantitative ultrasound assessment in clinical decision-making. In the present case, Modified Heckmatt Scale findings were consistent with Grade 2 changes, suggesting early-to-moderate non-neural muscle alterations characterized by increased echogenicity and partial architectural disruption rather than advanced structural degeneration. Nevertheless, persistent pain, heterogeneous muscle appearance, and functional limitations indicated that non-neural musculoskeletal factors remained clinically relevant. Consequently, adjunctive therapies such as ESWT and NMES were incorporated not because of a specific ultrasound grade alone, but because ultrasound findings were interpreted within the broader clinical context. This observation reinforces the principle that musculoskeletal ultrasound should complement, rather than replace, comprehensive clinical assessment when selecting individualized multimodal interventions.

### 4.4. Study Limitations and Future Directions

This study presents a proof-of-concept clinical framework illustrated through a single representative case, which inherently limits the generalizability of the findings and precludes conclusions regarding treatment efficacy or causal relationships. The objective was not to evaluate the efficacy of a specific treatment strategy but to demonstrate the feasibility of integrating serial musculoskeletal ultrasound, standardized clinical assessment, BoNT-A, complementary therapies, and comprehensive neurorehabilitation within a structured clinical decision-making pathway. As such, the illustrative case should be interpreted as an example of framework implementation rather than evidence of the effectiveness of a specific treatment protocol.

Although conventional B-mode musculoskeletal ultrasound provides valuable information regarding muscle architecture, echogenicity, and structural muscle quality, it cannot fully characterize changes in tissue stiffness, extracellular matrix remodeling, or muscle mechanical properties. Future prospective studies involving larger, clinically representative patient cohorts are required to validate the proposed framework, determine its reproducibility and generalizability, and evaluate its potential impact on clinical decision-making and patient outcomes. In addition, future research should investigate whether systematic serial musculoskeletal ultrasound assessment, incorporating semi-quantitative tools such as the Modified Heckmatt Scale, can improve longitudinal monitoring of the non-neural component of the post-stroke spasticity syndrome, identify patients at risk of progressive muscle deterioration, and facilitate earlier implementation of individualized multimodal interventions. Such an approach may contribute to more personalized treatment strategies, optimize long-term functional outcomes, and further establish musculoskeletal ultrasound as a practical clinical decision-support tool. The feasibility of acquiring serial ultrasound images during routine treatment sessions also suggests a potential role for ultrasound in documenting longitudinal muscle status and supporting quality assurance within specialized spasticity management programs. As imaging technologies continue to evolve, future studies should investigate the complementary role of quantitative techniques, including ultrasound elastography, diffusion tensor imaging, and biomarkers of extracellular matrix remodeling, to better characterize treatment-induced muscle adaptations and further validate imaging-informed multimodal rehabilitation strategies.

Taken together, this proof-of-concept framework does not establish treatment efficacy but provides a practical foundation for future prospective validation studies integrating both neural and non-neural components of the post-stroke spasticity syndrome.

## 5. Conclusions

Post-stroke spasticity is a dynamic and multidimensional syndrome resulting from the interaction of neural hyperexcitability and non-neural musculoskeletal adaptations. Effective management therefore requires treatment strategies that extend beyond the reduction in muscle tone alone and address the broader functional consequences of spasticity. This article proposes a practical, ultrasound-informed clinical framework that integrates serial musculoskeletal ultrasound, standardized clinical assessment, botulinum toxin type A (BoNT-A), adjunctive physical modalities, and task-oriented neurorehabilitation within a structured decision-making pathway.

The illustrative case demonstrates the feasibility of applying this framework in clinical practice and highlights how serial musculoskeletal ultrasound may complement clinical evaluation by supporting muscle targeting, treatment planning, and longitudinal monitoring. Although favorable changes in spasticity, pain, mobility, motor performance, and functional independence were observed during follow-up, these findings should be interpreted as illustrative of the proposed framework rather than as evidence of treatment efficacy. The modest changes in muscle morphology observed on ultrasound, despite clinical improvement, further emphasize the importance of interpreting imaging findings within the context of comprehensive clinical assessment.

As a proof-of-concept illustrated through a single representative case, the proposed framework highlights the potential value of integrating musculoskeletal ultrasound into multimodal spasticity management and supports its role as a practical clinical decision-support tool. Future prospective studies involving larger patient cohorts are warranted to validate the framework, determine its reproducibility and generalizability, and further investigate the role of musculoskeletal ultrasound as a biomarker for individualized rehabilitation planning.

## Figures and Tables

**Table 1:** Summary of the proposed ultrasound-informed multimodal clinical framework for the assessment, treatment planning, intervention, and longitudinal monitoring of post-stroke spasticity.

Step	Assessment/Intervention	Purpose Within the Framework
Initial clinical assessment	MAS, PROM, VAS, FAC, FMA-UE, FMA-LE, TUG	Characterize neural and functional impairment
Baseline MSK ultrasound	Muscle architecture, echogenicity, MHS	Assess non-neural muscle changes
Treatment planning	Clinical + ultrasound findings	Select muscles for BoNT-A and adjunctive therapies
Multimodal intervention	BoNT-A, NMES, ESWT, task-oriented rehabilitation	Address neural and non-neural components
Follow-up assessment	Repeat clinical assessment + serial MSK US	Monitor response and adapt treatment

Abbreviations: BoNT-A: Botulinum toxin type A; ESWT: extracorporeal shock wave therapy; FMA-LE: Fugl–Meyer Assessment for Lower Extremity; FMA-UE: Fugl–Meyer Assessment for Upper Extremity; MAS: Modified Ashworth Scale; MHS: Modified Heckmatt Scale; MSK US: musculoskeletal ultrasound; NMES: neuromuscular electrical stimulation; PROM: passive range of motion; TUG: Timed Up and Go Test; VAS: Visual Analogue Scale.

**Table 2:** Patient characteristics.

	Value
Age (years)	52
Weight (kg)	84.52
Height (cm)	179
Time since stroke onset (months)	24.02
Gender (M/F)	M
Stroke type (Ischemic/Hemorrhagic)	Hemorrhagic
Affected side of body (Right/Left)	Right

Abbreviations: M/F: male/female.

**Table 3:** Integration of ultrasound findings into treatment planning within the proposed framework and clinical assessment across the rehabilitation program.

Outcome Measures	T0	T1 (3-Month)	Clinical Interpretation
MAS UE and LE	3	2	Reduced muscle tone
Knee PROM (degrees)	120	125	Augmented mobility
Ankle PROM (degrees)	43	47	Augmented mobility
VAS	7	4	Decrease in pain intensity
FAC	3	4	Increased level of ambulation
Tinetti Assessment Tool	14	22	Improved balance and gait
FMA-UE	21	24	Improved motor function, coordination
FMA-LE	22	26	Improved motor function, coordination
TUG (seconds)	31.02	25.09	Improved functional mobility
**MSK** **-clinical findings**	**Ultrasound findings**	**Clinical findings**	**Treatment decision and rationale**
Baseline	Altered echotexture and increased stiffness	MAS 3 and FAC 3 limited balance and gait	Neural overactivity and structural changes; BoNT-A injection in spastic muscles
Post-BoNT-A (early phase)	Reduced dynamic muscle overactivity appearance	Decreased MAS degree	Facilitates muscle activation and functional integration; Initiation of ES and ESWT due to additional increased VAS
Follow-up (3 months)	Slightly modified muscle morphology on serial MSK imaging	Improved TUG, FAC	Adaptive management based on reassessment Continued rehabilitation; Injection planning
**MSK US Parameter**	**T0**	**T1**	Presence of non-neural muscular involvement (increased echogenicity and altered muscle quality) supported inclusion of ESWT and NMES within the multimodal rehabilitation program
Modified Heckmatt Scale (MHS) Score	**2**	**2**	

Abbreviations: BoNT-A: Botulinum toxin type A; ES: electrical stimulation; ESWT: extracorporeal shock wave therapy; FMA-LE: Fugl–Meyer Assessment for Lower Extremity FMA-UE: Fugl–Meyer Assessment for Upper Extremity; MAS: Modified Ashworth Scale; MSK: musculoskeletal; PROM: passive range of motion; TUG: Timed Up and Go Test; VAS: Visual Analogue Scale. T0: baseline assessment; T1: follow-up at 3 months.

**Table 4:** Botulinum toxin type A injection strategy applied in the illustrative case.

Muscle	Injected Dose (U)	Guidance
Pectoralis major	100	MSK US
Brachialis	100	MSK US
Biceps brachii	100	MSK US
Flexor superficialis digitorum	100	MSK US
Flexor carpi radialis	100	MSK US
Soleus	100	MSK US
Gastrocnemius both heads	75 and 75	MSK US
Tibialis posterior	100	MSK US

Abbreviations: MSK US: Musculoskeletal Ultrasound.

## Data Availability

Data are contained within the article.

## Appendix A. Ultrasound-Informed Clinical Decision-Making Pathway

Figure A1 provides a practical clinical decision-making algorithm that complements the proposed ultrasound-informed multimodal framework. While Figure 1 summarizes the overall workflow of the framework, Figure A1 illustrates how standardized clinical assessment and serial musculoskeletal ultrasound findings can be integrated to support individualized treatment selection, longitudinal monitoring, and treatment adaptation in patients with post-stroke spasticity. The algorithm emphasizes the complementary evaluation of neural and non-neural contributors to spasticity and the iterative use of clinical reassessment to optimize multimodal rehabilitation.
